# Point-of-care Tests for *Chlamydia trachomatis* and *Neisseria gonorrhoeae*: Review of the Literature

**DOI:** 10.1093/cid/ciaf699

**Published:** 2026-02-24

**Authors:** Yukari C Manabe, Amanda C Smith, Susan Trow, Anthony Tran, Barbara Van Der Pol

**Affiliations:** Department of Medicine, Division of Infectious Diseases, Johns Hopkins School of Medicine, Baltimore, Maryland, USA; Division of STD Prevention, National Center for HIV, Viral Hepatitis, STD, and TB Prevention, US Centers for Disease Control and Prevention, Atlanta, Georgia, USA; Association of Public Health Laboratories, Bethesda, Maryland, USA; California Department of Public Health, Center for Laboratory Sciences, Richmond, California, USA; Department of Medicine, Division of Infectious Diseases, University of Alabama at Birmingham Heersink School of Medicine, Birmingham, Alabama, USA

**Keywords:** point-of-care tests, gonorrhea, chlamydia, review

## Abstract

**Background:**

Point-of-care tests (POCTs) for the detection of *Chlamydia trachomatis* and *Neisseria gonorrhoeae* offer the potential for rapid diagnosis and treatment, improving patient outcomes and reducing disease transmission. We sought to compile evidence on the accuracy and clinical utility of molecular-based POCTs and near-POCTs for the detection of *C. trachomatis* and *N. gonorrhoeae*.

**Methods:**

We performed a systematic literature search of 5 electronic databases from January 2009 to January 2024 to understand the performance characteristics and implementation considerations associated with Food and Drug Administration–cleared POCTs and near-POCTs. Results were described in a narrative format.

**Results:**

From 3743 identified studies, 64 met our inclusion criteria. As of 2025, there are 4 Food and Drug Administration–cleared POCTs/near-POCTs for detecting *C. trachomatis* and *N. gonorrhoeae*, 3 of which are waived by the Clinical Laboratory Improvement Amendments and suitable for use during a patient visit. Evidence suggests that POCTs are most beneficial in symptomatic patients within acute care settings, where they can prevent loss to follow-up and reduce the need for empiric antibiotic treatment.

**Conclusions:**

While some POCTs for *C. trachomatis* and *N. gonorrhoeae* have achieved regulatory clearance, challenges remain, including the need to expand specimen type clearance to include extragenital specimens, to further improve turnaround times, and to decrease cost for adoption. There is need to optimize the use of POCTs in acute care settings to manage sexually transmitted infections.

The Centers for Disease Control and Prevention’s (CDC’s) 2024 provisional sexually transmitted infection (STI) surveillance identified >2.0 million combined cases of chlamydia and gonorrhea in the United States (US) [1]; in Europe, increases in reported STI cases in 2023 compared with 2022 were noted [2]. While healthcare disruptions related to the coronavirus disease 2019 (COVID-19) pandemic have stabilized, reported chlamydia and gonorrhea cases have declined in the US compared with the previous year [3]. However, health disparities continue to persist and increase, with 48.2% of chlamydia, gonorrhea, and syphilis cases occurring in adolescents and young adults (aged 15–24 years) and 34.8% in non-Hispanic blacks and African Americans. Despite the decreased availability and underfunding of STI clinics over the past decade [1], access to testing services has expanded in other settings, including primary care settings. Strategies to improve testing include guidelines instructing the routine testing of all sexually active women <25 years old [4, 5], increased testing by primary healthcare providers, Food and Drug Administration (FDA) authorization of self-collection for most reference laboratory testing platforms, remote testing [6], and point-of-care tests (POCTs).

Reference laboratory testing for *Neisseria gonorrhoeae* and *Chlamydia trachomatis* is usually initiated by healthcare providers and usually occurs as a dual-multiplexed test. Nucleic acid amplification tests (NAATs) have excellent sensitivity (>95%) and specificity (>98%), with turnaround times that range from 24 to 72 hours in most settings. However, the time to result varies depending on where the testing will be done relative to where the specimen is collected. Historically, clinician-collected cervical swab or urethral swab samples were the recommended sample types due to reliance on culture-based methods [7]. However with the widespread use of NAATs, meta-analyses have demonstrated the superiority of vaginal swab (VS) over urine samples [8], the equivalence of self-collected to clinician-collected VS samples, and the effectiveness of first-void urine for men in STI detection [9]. Therefore, self-collected VS and urine samples are the recommended and preferred sample types for detecting urogenital *C. trachomatis* and *N. gonorrhoeae* in women and men, respectively [5, 10]. These findings underscore the shift toward more patient-centered, noninvasive sampling methods that maintain or improve diagnostic performance.

Building on the advancements in patient-centered care, efforts have increasingly focused on improving accessibility and timely diagnosis through POCTs. The FDA classifies Clinical Laboratory Improvement Amendments (CLIA)­–waived POCTs as “simple laboratory procedures that have an insignificant risk of an erroneous result” when performed by nonlaboratorians following the instructions for use. True POCTs can be performed at the point of care within a single patient visit. In contrast, non–CLIA-waived near-POCTs must be performed in a CLIA-regulated moderate- or high-complexity laboratory. The COVID-19 pandemic accelerated POCT development capable of providing results within a single clinical encounter, as numerous tests received emergency use authorization [11]. POCTs for STIs could expand access to testing in underserved and rural populations while enabling rapid diagnosis and expedited treatment, which are critical for interrupting transmission, particularly in periodic acute care settings where follow-up may be challenging (eg, emergency departments). This review compiles evidence on the accuracy and clinical utility of molecular-based POCTs and near-POCTs for the detection of *C. trachomatis* and *N. gonorrhoeae* and identifies key research gaps.

## METHODS

To identify relevant literature using an unbiased approach for our systematic literature search and narrative review, the CDC initiated a literature search through a CDC Stephen B. Thacker librarian. The results of the request were returned on 1/18/2024. Databases included Medline (OVID), Embase (OVID), Cochrane Library, CINAHL (EBSCOHost), and Scopus. The publication date range was limited to January 2009 to January 2024, limiting publications to POCTs/near-POCTs in use during this period. A broad scope was selected for the search, targeting retrieval of as many citations as possible. Search terms included but were not limited to “chlamydia OR chlamydia trachomatis OR gonor* OR neisseria gonor* point-of-care OR rapid AND test OR POC OR diagnostic test”. A full list of search terms can be found in Supplementary File 1.

To be included in the review, the articles had to meet the following criteria: clinical studies that were published in English in peer-reviewed journal articles and that uses a POCT or near-POCT for diagnostic testing of chlamydia and gonorrhea. Additional information was obtained from package inserts from FDA approvals and clearances.

The following criteria were used to identify and narrow the literature: population, including all sexually active individuals, with no age restrictions; the intervention, including any molecular POCT or near-POCT is cleared by the FDA within the United States; comparison with the standard of care, typically a laboratory-based FDA-approved NAAT; and outcome, with a primary focus on test performance metrics (eg, sensitivity and specificity). Secondary outcomes included patient-centered metrics, such as time to treatment and the appropriate use of antibiotics.

**Figure 1.**
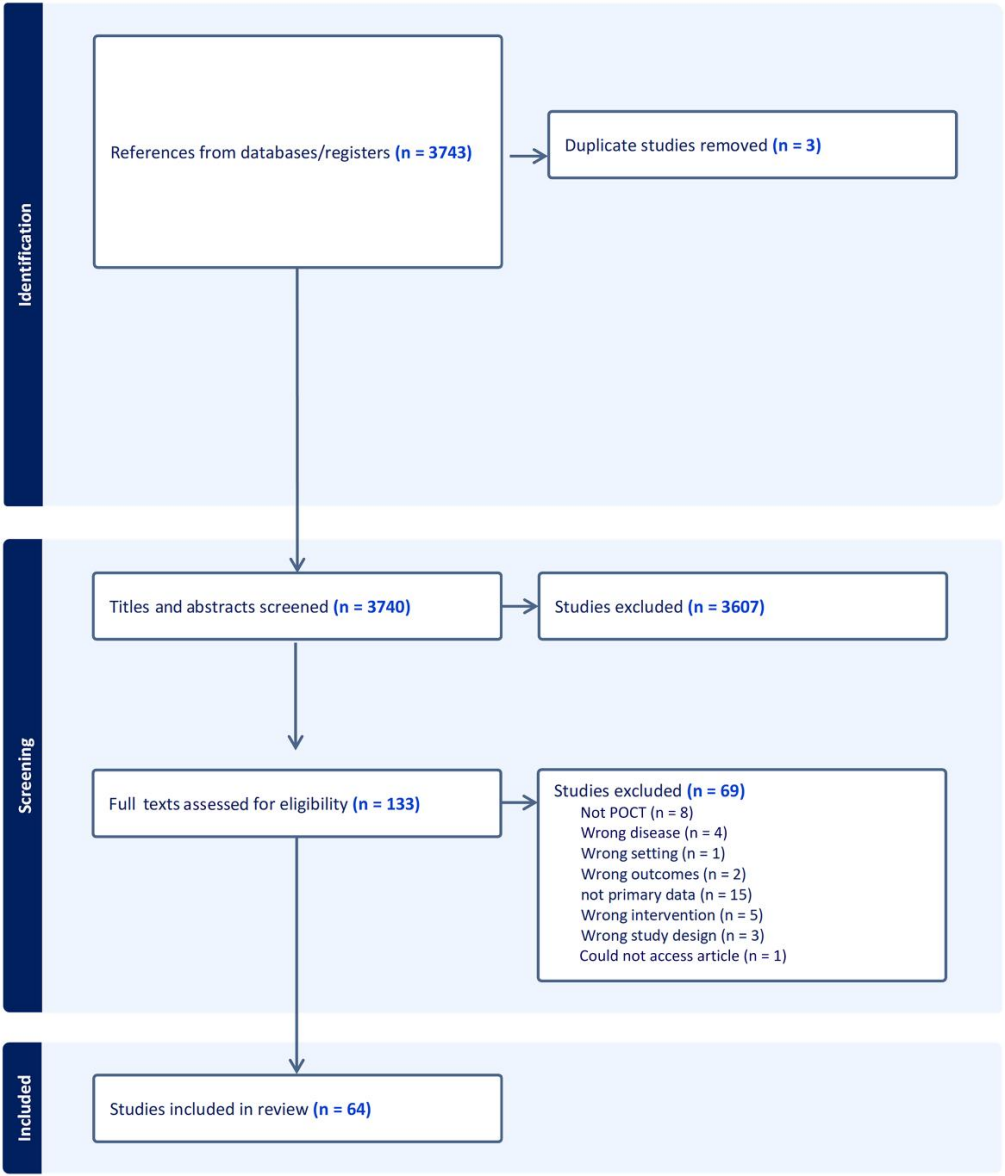
PRISMA diagram for the Preferred Reporting Items for Systematic Reviews and Meta-Analyses flowchart of the publication inclusion process. Abbreviation: POCT, point-of-care test.

Overall, the literature search identified 3743 publications, which were imported into the Covidence online platform for systematic review management. Following the removal of duplicated publications (n = 3), reviewers screened 3740 titles and abstracts for inclusion. Only 133 publications met inclusion criteria based on the title and abstract alone. Of those publications, 69 were excluded, leaving 64 studies for abstraction (Figure 1). The authors developed and used a standardized form to abstract data from all the included studies which included the study aim, description of test methods used, setting and population description, key results, and strengths and weaknesses of the study. Data were abstracted by B. V. D. P., Y. C. M., or A. T. in Covidence (Covidence systematic review software; Veritas Health Innovation). The data were exported to a table and information reviewed by A. S. or S. T. for accuracy. After data abstraction, studies were divided into 5 main categories: (1) FDA-cleared tests—performance characteristics; (2) FDA-cleared tests—use characteristics; (3) tests that have not advanced or were obsolete; (4) emerging tests not yet cleared by the FDA; and (5) reviews/editorials.

## RESULTS

### Performance of FDA-Cleared Point-of-care NAAT Technologies

Currently, there are 4 FDA-cleared POCTs/near-POCTs for the detection of *C. trachomatis* and *N. gonorrhoeae* (Table 1). Strong analytical performances have been demonstrated for all FDA-cleared tests (Table 2 and Supplementary Table 1). The binx io CT/NG (binx health), Visby Sexual Health Test (Visby Medical) and the cobas liat CT/NG/MG (Roche Diagnostics) nucleic acid test are all CLIA-waived tests with a <30-minute turnaround time, whereas the Xpert CT/NG assay (Cepheid), a 90-minute, moderately complex near-POCT, has not yet achieved CLIA-waived status despite simple operating characteristics. All tests provide results for *N. gonorrhoeae* and *C. trachomatis*; the Visby test also generates a *Trichomonas vaginalis* result, and the cobas liat test also generates a *Mycoplasma genitalium* result.

**Table 1.** **Food and Drug Administration–Cleared Point-of-care and Near–Point-of-care Tests for *Neisseria gonorrhoeae* and *Chlamydia trachomatis*** ^a^

Assay	Ages Evaluated, y	Turnaround-Time, min	Throughput	CLIA-Waived	Accepted Specimen Types	Reference(s)
Cepheid Xpert CT/NG Xpert CT/NG (https://www.cepheid.com/content/dam/www-cepheid-com/documents/package-insert-files/xpert-ct-ng-(english)-genexpert-system-with-touchscreen/Xpert%20CTNG%20US%20ENGLISH%20IFU%20303-0941%20Rev%20%20A.pdf; cepheid.com)	≥14 (F and M)	90	Single cartridge per module; multiple module systems with random access	No	Urine (F and M); patient-collected VS, clinician-collected ES; clinician-collected pharyngeal and rectal swab (F and M)	[12–29]
Visby Medical Sexual Health test (*Chlamydia trachomatis*, *Neisseria gonorrhoeae*, and *Trichomonas vaginalis* ) (https://www.visbymedical.com/assets/sexual-health-test/Visby-Medical-Sexual-Health-Test-Instructions-for-Use.pdf)	≥14 (F)	<30	Single-use throwaway test	Yes	Patient-collected VS	[30]
binx IO CT/NG (MOB_ART_0700_Black_White_Single_Pages_8374a31021.pdf)	≥16 (F); ≥17 (M)	<30	Single cartridge per module	Yes	Clinician and patient-collected VS; urine (M)	[31–33]
Roche cobas liat CT/NG/MG or cobas liat CT/NG (https://www.accessdata.fda.gov/cdrh_docs/clia_waivers/CW240002.pdf)	≥18 (F and M)	20	Single sample	Yes	Clinician and patient-collected VS; urine (M)	[34]

Abbreviations: CLIA, Clinical Laboratory Improvement Amendments; ES, endocervical swab; F, females; FDA, Food and Drug Administration; M, males; VS, vaginal swab.

^a^Sensitivity and specificity or positive and negative percent agreement were referenced directly from the instructions for use for each assay. Positive and negative percent agreement were calculated for the Visby Medical Sexual Health Test and for VS samples with the cobas liat CT/NG/MG.

**Table 2.** Assay Performance of Food and Drug Administration–Cleared Point-of-care and Near–Point-of-care Tests

Assay	Sensitivity/PPA, % (95% confidence intervals)	Specificity/NPA, % (95% confidence intervals)
Cepheid Xpert CT/NG (https://www.cepheid.com/content/dam/www-cepheid-com/documents/package-insert-files/xpert-ct-ng-(english)-genexpert-system-with-touchscreen/Xpert%20CTNG%20US%20ENGLISH%20IFU%20303-0941%20Rev%20%20A.pdf; cepheid.com)	VS sample for *Chlamydia trachomatis* (F): symptomatic, 100 (95.4–100); asymptomatic, 99.2 (95.5–100)	VS sample for *C. trachomatis* (F): symptomatic, 98.4 (97.5–99.0); asymptomatic, 99.5 (99.2–99.8)
	ES sample for *C. trachomatis* (F): symptomatic, 96.2 (89.3–99.2); asymptomatic, 95.9 (90.7–98.7)	ES sample for *C. trachomatis* (F): symptomatic, 99.6 (99.0–99.9); asymptomatic, 99.5 (99.2–99.8)
	Urine sample for *C. trachomatis* (F): symptomatic, 98.8 (93.6–100); asymptomatic, 97.6 (93.2–99.5)	Urine sample for *C. trachomatis* (F): symptomatic, 99.7 (99.2–99.9); asymptomatic, 99.9 (99.7–100)
	Urine sample for *C. trachomatis* (M): symptomatic, 97.6 (93.0–99.5); asymptomatic, 100.0 (95.1–100)	Urine sample for *C. trachomatis* (M): symptomatic, 99.7 (98.8–100); asymptomatic, 99.8 (99.6–99.9)
	Pharyngeal swab sample for *C. trachomatis*: symptomatic, 100.0 (70.1–100.0); asymptomatic, 95.0 (83.5–98.6)	Pharyngeal swab sample for *C. trachomatis*: symptomatic, 100.0 (98.7–100.0); asymptomatic, 99.6 (99.3–99.8
	Rectal swab sample for *C. trachomatis*: symptomatic, 81.5 (63.3–91.8); asymptomatic, 86.6 (81.3–90.7)	Rectal swab sample for *C. trachomatis*: symptomatic, 99.4 (96.6–99.9); asymptomatic, 99.4 (98.9–99.6)
	VS sample for *Neisseria. gonorrhoeae* (F): symptomatic, 100 (87.2–100); asymptomatic, 100 (86.3–100)	VS sample for *N. gonorrhoeae* (F): symptomatic, 99.8 (99.4–100); asymptomatic, >99.9 (99.8–100)
	ES sample for *N. gonorrhoeae* (F): symptomatic, 100 (87.2–100); asymptomatic, 100 (86.3–100)	ES sample for *N. gonorrhoeae* (F): symptomatic, 99.9 (99.6–100); asymptomatic, 100 (99.8–100)
	Urine sample for *N. gonorrhoeae* (F): symptomatic, 96.6 (82.2–99.9); asymptomatic, 92.0 (74.0–99.0)	Urine sample for *N. gonorrhoeae* (F): symptomatic, 100 (99.7–100); asymptomatic, >99.9 (99.8–100)
	Urine sample for *N. gonorrhoeae* (M): symptomatic, 99.1 (94.9–100); asymptomatic, 92.3 (64.0–99.8)	Urine sample for *N. gonorrhoeae* (M): symptomatic, 100 (99.4–100); asymptomatic, 99.9 (99.7–100)
	Pharyngeal swab sample for *N. gonorrhoeae*: symptomatic, 92.9 (81.0–97.5); asymptomatic, 95.1 (90.7–97.5)	Pharyngeal swab sample for *N. gonorrhoeae*: symptomatic, 98.9 (96.7–99.6); asymptomatic, 98.8 (98.2–99.2)
	Rectal swab sample for *N. gonorrhoeae*: symptomatic, 97.4 (86.8–99.6); asymptomatic, 89.8 (84.2–93.5)	Rectal swab sample for *N. gonorrhoeae*: symptomatic, 99.4 (96.6–99.9); asymptomatic, 99.4 (98.9–99.6)
Visby Medical Sexual Health test (*C. trachomatis*, *N. gonorrhoeae*, and *Trichomonas vaginalis*) (https://www.visbymedical.com/assets/sexual-health-test/Visby-Medical-Sexual-Health-Test-Instructions-for-Use.pdf; visbymedical.com)	*C. trachomatis*: symptomatic, 97.9 (92.8–99.4); asymptomatic, 96.4 (87.9–99.0)	*C. trachomatis*: symptomatic, 96.8 (95.4–97.8); asymptomatic, 98.8 (97.7–99.3)
	*N. gonorrhoeae*: symptomatic, 100 (86.7–100); asymptomatic, 95.0 (76.4–99.1)	*N. gonorrhoeae*: symptomatic, 99.1 (98.3–99.6); asymptomatic, 99.0 (98.1–99.5)
binx IO CT/NG (MOB_ART_0700_Black_White_Single_Pages_8374a31021.pdf)	VS sample for *C. trachomatis* (F): symptomatic, 95.2 (86.7–98.3); asymptomatic, 97.0 (89.8–99.2)	VS sample for *C. trachomatis* (F): symptomatic, 98.9 (97.9–99.5); asymptomatic, 99.2 (98.2–99.7)
	VS sample for *N. gonorrhoeae* (F):symptomatic, 100 (88.3–100); asymptomatic, 100 (80.6–100)	VS sample for *N. gonorrhoeae* (F): symptomatic, 99.9 (99.3–100); asymptomatic, 100 (89.2–100)
	Urine sample for *C. trachomatis* (M): symptomatic, 91.7 (81.9–96.4); asymptomatic, 93.3 (89.8–99.2)	Urine sample for *C. trachomatis* (M): symptomatic, 99.6 (97.8–99.9); asymptomatic, 99.1 (97.9–99.6)
	Urine sample for *N. gonorrhoeae* (M): symptomatic, 98.4 (91.4–99.7); asymptomatic, 91.7 (64.6–98.5)	Urine sample for *N. gonorrhoeae* (M): symptomatic, 100 (98.5–100); asymptomatic, 100 (98.6–100)
cobas liat CT/NG/MG (https://www.accessdata.fda.gov/cdrh_docs/clia_waivers/CW240002.pdf)	VS sample for *C. trachomatis* (F): symptomatic, 98.4 (91.3–99.7); asymptomatic, 97.9 (89.1–99.7)	VS sample for *C. trachomatis* (F): symptomatic, 99.7 (99.2–99.9); asymptomatic, 99.8 (99.4–100)
	VS sample for *N. gonorrhoeae* (F): symptomatic, 95.5 (84.9–98.7); asymptomatic, 100 (91.8–100)	VS sample for *N. gonorrhoeae* (F): symptomatic, 99.8 (99.3–100);asymptomatic, 99.9 (99.5–100)
	Urine sample for *C. trachomatis* (M): symptomatic, 98.2 (90.6–99.7); asymptomatic, 96.4 (87.7–99.0)	Urine sample for *C. trachomatis* (M): symptomatic, 99.9 (99.3–100); asymptomatic, 99.9 (99.2–99.9)
	Urine sample for *N. gonorrhoeae* (M): symptomatic, 100 (97.4–100); asymptomatic, 100 (80.6–100)	Urine sample for *N. gonorrhoeae* (M): symptomatic, 100 (99.5–100); asymptomatic, 99.8 (99.4–100)

Abbreviations: ES, endocervical swab; F, females; M, males; NPS, negative percent agreement; PPS, positive percent agreement; VS, vaginal swab.

Multiple studies and systematic reviews have reported that the Xpert CT/NG assay achieves sensitivity >95% and specificity >99% [12]. In a company-sponsored study involving >1400 patients, the Visby Sexual Health Test demonstrated high sensitivity (>97%) and specificity (>97%) compared with laboratory-based NAATs. However, no male sample type (eg, first-void urine or penile meatal swab sample) has been cleared for use with this device to date [30]. The binx io CT/NG test also showed excellent sensitivity and specificity in a large multisite study. However, the sensitivity of male urine samples for chlamydia detection fell below the target performance threshold of 95%, with a lower 95% confidence interval of 90% [31, 32]. Most recently, the FDA-cleared cobas liat demonstrated >95% sensitivity and specificity for chlamydia and gonorrhea detection in male urine samples and VS samples in a large multisite study involving >4800 patients [34].

These assays vary with regard to approved specimen type and turnaround time (Table 1 and Supplementary Table 1). Among CLIA-waived tests, the Visby test is approved only for self-collected VS samples, whereas the binx io and cobas liat tests can be used with both male first-void urine and self-collected VS samples. The Xpert CT/NG is the only FDA-cleared near-POCT with claims for extragenital sample types, but it must be performed in a moderate-complexity laboratory and takes approximately 90 minutes [13]. However, international studies have evaluated the use of the Xpert CT/NG assay for extragenital POCTs in clinical settings [14].

### Clinical Use Case: When to Use POCTs

In the United States, the best-supported use case for *N. gonorrhoeae*/*C. trachomatis* POCTs is among symptomatic patients in acute care settings, where same-visit results can guide treatment and reduce loss to follow-up [28, 35]. A study of 55 women using the Visby device in a periodic acute care setting demonstrated that POCTs helped reduce antibiotic overuse [36]. The test correctly identified 13 of 15 undertreated patients as infected and accurately detected all 33 cases of overtreatment. Clinicians valued the rapid results, and patients were comfortable waiting up to 30 minutes. Similarly, in a small randomized controlled trial of the Xpert CT/NG assay in an emergency department, patients who received negative near-POCT results (8 of 37 [21.6%]) were less likely to be given empirical antibiotics than the control group (11 of 20 [55%]) [37].

This finding was reinforced by a pragmatic quasi-experimental study in an urban emergency department, which also reported a reduction in antibiotic overuse and significantly shorter emergency department stays [38]. An implementation study of the Xpert CT/NG assay at a high-volume suburban urgent care clinic further supported its benefits. Compared with a historical control group receiving traditional urogenital *N. gonorrhoeae*/*C. trachomatis* laboratory-based testing, the near-POCT improved treatment appropriateness. Empirical overtreatment occurred in 40% of patients with traditional testing (n = 40) but was eliminated in the POCT group (*P* < .001). Similarly, undertreatment was observed in 8% of the traditional group (n = 8) but was absent in the POCT group (*P* = .004). The POCT approach also achieved cost neutrality (excluding instrument costs and service agreements) and was associated with high staff satisfaction [35].

Patient willingness to wait for POCT results is another critical factor. In a large multisite study of the binx io, the 30-minute POCT successfully delivered results to patients who were unwilling to wait >90 minutes for traditional testing [31]. In 2 additional studies—one among adolescents and another among university students—61% and 89.4% of participants, respectively, were willing to wait up to 20 minutes for their results [39, 40]. Cordioli et al [14] further reported that most patients (95.95% [n = 1633]) were open to POCTs and willing to wait on site for results, though wait-time tolerance varied: 22.4% would wait up to 2 hours, 41.6% up to 1 hour, and 31% up to 30 minutes.

Other use cases might include remote rural settings where laboratory access is limited and turnaround times are longer. There is a lack of US data to evaluate for this context; however, several studies from Australia demonstrated the acceptability and feasibility of this scheme [23, 41]. Additional factors—such as instrument size, throughput, refrigeration, and power requirements—may influence the feasibility of integrating these systems into clinical workflows. Currently, only binx io and the GeneXpert platform have the capability to connect with laboratory information systems or electronic medical records, facilitating streamlined data capture.

### Future Directions and Emerging Tests

Despite the excellent performance of the 4 FDA-cleared POCTs/near-POCTs, STI POCTs remain relatively new, with several platforms in development. The Cepheid Xpert Xpress, a CLIA-waived 60-minute test for urogenital *C. trachomatis*/*N. gonorrhoeae*, is under study, with ongoing efforts to expand clearance for extragenital specimens. Research is also focused on integrating resistance detection into POCTs. The PROMPT test, a 20-minute assay for detecting ciprofloxacin resistance (*gyrA* mutations), is in development [42]. In addition, the Antibacterial Resistance Leadership Group has initiated the Multiple Infection Diagnostics Resistant *Neisseria gonorrhoeae* (MASTERMIND-RING) study to evaluate the diagnostic accuracy of a reflex test on urogenital and pharyngeal specimens to detect the DNA gyrase mutation (gyrA S91F).

While previous lateral flow assays for *C. trachomatis*/*N. gonorrhoeae* had poor performance [43–46], a new *N. gonorrhoeae*–specific lateral flow assay in development demonstrated >90% sensitivity and >95% specificity for both urine and VS specimens, with a run time of <30 minutes [47, 48]. As a lateral flow assay, the test is likely to be highly cost-effective, though retail pricing is yet to be established. Its low cost may be an advantage, but its clinical utility remains uncertain since providers would still need to empirically treat *C. trachomatis*.

The growing landscape of over-the-counter (OTC) STI tests also affects the role of POCTs. Let's Get Checked received marketing authorization in late 2023 for their OTC home self-collection kit for *C. trachomatis*/*N. gonorrhoeae* laboratory testing on the Aptima Combo 2 (Hologic). In addition, Visby received marketing authorization from the FDA in March 2025 for the first 30-minute OTC polymerase chain reaction–based self-test for *C. trachomatis*/*N. gonorrhoeae*/*T. vaginalis* using VS samples. If affordably priced, it could increase STI testing and reduce transmission, though questions remain about result reporting and linkage to care at this point.

## DISCUSSION

Several important implementation considerations are worth highlighting. First, POCTs may be expensive for asymptomatic screening as this is not reimbursable through the Centers for Medicare & Medicaid Services in 2025, even though the United States Preventive Services Task Force recommends screening for chlamydia and gonorrhea in all sexually active women ≤24 years old and in women ≥25 years old who are at increased risk for infection (grade B) [4]. In addition, some of the platforms have costly service contracts that have volume-based implications. In remote settings where follow-up can be challenging, the rapid linkage to treatment may outweigh the additional cost compared with reference laboratory testing. Historically, reference CLIA-licensed laboratories have reported notifiable STIs; although clinicians are mandated to report positive results in most jurisdictions, gaps in reporting exist. The variability in the way that data are captured, and results communicated to the electronic medical record may affect the number of notifiable infections that are reported to public health agencies.

Although quarterly 3-site STI screening is recommended for all patients receiving HIV preexposure prophylaxis [5], none of the existing CLIA-waived platforms currently have extragenital claims. In addition, none of the laboratory-based platforms have self-collected rectal or throat claims either, so clinical studies for POCTs using extragenital specimens will not have a clear comparator, which will add additional complexity and cost to the process of obtaining FDA regulatory approval. Finally, the ways in which commercially available tests are currently multiplexed may not align with recommendations for use in target populations for future claims (eg, inclusion of *T. vaginalis* in approved POCTs such as the Visby test for non-VS samples and inclusion of *M. genitalium* in the Roche liat test). However, this issue is not exclusive to POCTs but also affects laboratory-based tests. Several newer platforms, such as the BD Max, also expand multiplex STI testing to include *T. vaginalis*.

Because CLIA-waived POCTs are generally simple to perform and have a low risk of result misinterpretation, considerations for quality management and proper training are often overlooked. Despite the lower complexity, errors in testing may result from lack of appropriately trained and competent staff along with appropriate continual quality assurance methodologies such as proficiency testing. Fluctuations in environmental conditions and improper handling of equipment and assay reagents may also lead to erroneous results. In addition, molecular-based tests have a high potential for contamination, which can affect patient results if not carefully controlled. Finally, many POCTs are disposable and generate a lot of biochemical and biohazardous waste. It is important to adhere to all federal and local regulations for medical and hazardous waste disposal.

A limitation of the current review is that there was no meta-analyses of the studies reviewed. Rather, the authors were tasked with individually reviewing a subset of the studies and providing feedback according to a rubric established by the Association of Public Health Laboratories and the CDC, as in a systematic review. Thus, the analyses provided represent a range of sensitivities, specificities, and limits of detection, as established by each of the studies rather than an independent analysis conducted separately by the authors. In addition, the dependence on published literature necessitates that the information contained herein may not be current at the time of publication, as this is rapidly evolving field.

The landscape of emerging OTC tests highlights the shifting role of POCTs in STI diagnostics. While linkage to care and public health reporting remain critical concerns for OTC testing, its potential to significantly increase overall access to testing cannot be overlooked, even if the system is imperfect. To maximize the impact of POCTs, their goals must be framed appropriately—not as a universal replacement for clinical visits but as a complementary tool to enhance decision making and expand access to diagnostics. As more POCT platforms gain regulatory clearance, they offer a promising avenue to increase testing rates. However, the gap between clinical guidelines and real-world application of POCTs must be addressed to ensure that these tools are used effectively and equitably in the broader public health context.

## Supplementary Material

ciaf699_Supplementary_Data

## Supplementary Data

Supplementary materials are available at *Clinical Infectious Diseases* online. Consisting of data provided by the authors to benefit the reader, the posted materials are not copyedited and are the sole responsibility of the authors, so questions or comments should be addressed to the corresponding author.

## References

[1] Centers for Disease Control and Prevention . Sexually transmitted infections surveillance, 2024. Available at: https://www.cdc.gov/sti-statistics/annual/index.html. Accessed 8 December.

[2] European Centre for Disease Prevention and Control . STI cases continue to rise across Europe. **2025**. Available at: https://www.ecdc.europa.eu/en/news-events/sti-cases-continue-rise-across-europe. Accessed 8 December.

[3] Alba C, Zheng Z, Wadhera RK. Changes in health care access and preventive health screenings by race and ethnicity. JAMA Health Forum 2024; 5:e235058-e. 38306093 10.1001/jamahealthforum.2023.5058 PMC10837752

[4] Davidson KW, Barry MJ, Mangione CM, et al Screening for chlamydia and gonorrhea: US preventive services task force recommendation statement. JAMA 2021; 326:949–56. 34519796 10.1001/jama.2021.14081

[5] Workowski KA, Bachmann LH, Chan PA, et al Sexually transmitted infections treatment guidelines, 2021. MMWR Recomm Rep 2021; 70:1–187. 10.15585/mmwr.rr7004a1 PMC8344968 34292926

[6] Smith AC, Thorpe PG, Learner ER, Galloway ET, Kersh EN. At-home specimen self-collection as an additional testing strategy for chlamydia and gonorrhoea: a systematic literature review and meta-analysis. BMJ Glob Health 2024; 9:e015349. 10.1136/bmjgh-2024-015349 PMC11404247 39191483

[7] Johnson RE, Newhall WJ, Papp JR, et al Screening tests to detect *Chlamydia trachomatis* and *Neisseria gonorrhoeae* infections–2002. MMWR Recomm Rep 2002; 51(RR-15):1–38. quiz CE1-4. 12418541

[8] Aaron KJ, Griner S, Footman A, Boutwell A, Van Der Pol B. Vaginal swab vs urine for detection of *Chlamydia trachomatis, Neisseria gonorrhoeae,* and *Trichomonas vaginalis*: a meta-analysis. Ann Fam Med 2023; 21:172–9. 36973065 10.1370/afm.2942 PMC10042575

[9] Lunny C, Taylor D, Hoang L, et al Self-collected versus clinician-collected sampling for chlamydia and gonorrhea screening: a systemic review and meta-analysis. PLoS One 2015; 10:e0132776. 26168051 10.1371/journal.pone.0132776 PMC4500554

[10] Centers for Disease Control and Prevention . Recommendations for the laboratory-based detection of *Chlamydia trachomatis* and *Neisseria gonorrhoeae*–2014. MMWR Recomm Rep 2014; 63(RR-02):1–19. PMC4047970 24622331

[11] Manabe YC . The impact of COVID-19 pandemic on technologic and process innovation in point-of-care diagnostics for sexually transmitted infections. Clin Biochem 2021; 117:75–83. 34808115 10.1016/j.clinbiochem.2021.11.003 PMC8604101

[12] Xie TA, Liu YL, Meng RC, et al Evaluation of the diagnostic efficacy of Xpert CT/NG for *Chlamydia trachomatis* and *Neisseria gonorrhoeae*. Biomed Res Int 2020; 2020:2892734. 33102576 10.1155/2020/2892734 PMC7576347

[13] Geiger R, Smith DM, Little SJ, Mehta SR. Validation of the GeneXpert® CT/NG assay for use with male pharyngeal and rectal swabs. Austin J HIV AIDS Res 2016; 3:1021. 27536736 PMC4985020

[14] Cordioli M, Gios L, Erbogasto A, et al Clinic-based evaluation of the dual Xpert CT/NG assay on the GeneXpert system for screening for extragenital chlamydial and gonococcal infections amongst men who have sex with men. BMC Infect Dis 2024; 24:224. 38418963 10.1186/s12879-024-09042-4 PMC10902931

[15] Footman A, Dionne-Odom J, Aaron KJ, Raper JL, Van Der Pol B. Performance of 4 molecular assays for detection of chlamydia and gonorrhea in a sample of human immunodeficiency virus-positive men who have sex with men. Sex Transm Dis 2020; 47:158–61. 32032316 10.1097/OLQ.0000000000001115 PMC7393578

[16] Badman SG, Bell SFE, Dean JA, et al Reduced sensitivity from pooled urine, pharyngeal and rectal specimens when using a molecular assay for the detection of chlamydia and gonorrhoea near the point of care. Sex Health 2020; 17:15–21. 31945307 10.1071/SH19028

[17] Gaydos CA . Review of use of a new rapid real-time PCR, the cepheid GeneXpert® (Xpert) CT/NG assay, for *Chlamydia trachomatis* and *Neisseria gonorrhoeae*: results for patients while in a clinical setting. Expert Rev Mol Diagn 2014; 14:135–7. 24450867 10.1586/14737159.2014.871495 PMC4061495

[18] Bristow CC, McGrath MR, Cohen AC, Anderson LJ, Gordon KK, Klausner JD. Comparative evaluation of 2 nucleic acid amplification tests for the detection of *Chlamydia trachomatis* and *Neisseria gonorrhoeae* at extragenital sites. Sex Transm Dis 2017; 44:398–400. 28604481 10.1097/OLQ.0000000000000627 PMC5486408

[19] Dize L, Silver B, Gaydos C. Comparison of the cepheid GeneXpert CT/NG assay to the Hologic Aptima Combo2 assay for the detection of *Chlamydia trachomatis* and *Neisseria gonorrhoeae* in self-collected rectal swabs. Diagn Microbiol Infect Dis 2018; 90:83–4. 29174733 10.1016/j.diagmicrobio.2017.10.013 PMC6125782

[20] Gaydos CA, Van Der Pol B, Jett-Goheen M, et al Performance of the cepheid CT/NG Xpert rapid PCR test for detection of *Chlamydia trachomatis* and *Neisseria gonorrhoeae*. J Clin Microbiol 2013; 51:1666–72. 23467600 10.1128/JCM.03461-12 PMC3716060

[21] Cosentino LA, Danby CS, Rabe LK, et al Use of nucleic acid amplification testing for diagnosis of extragenital sexually transmitted infections. J Clin Microbiol 2017; 55:2801–7. 28679521 10.1128/JCM.00616-17 PMC5648715

[22] Causer LM, Hengel B, Natoli L, et al A field evaluation of a new molecular-based point-of-care test for chlamydia and gonorrhoea in remote Aboriginal health services in Australia. Sex Health 2015; 12:27–33. 25426655 10.1071/SH14158

[23] Causer LM, Guy RJ, Tabrizi SN, et al Molecular test for chlamydia and gonorrhoea used at point of care in remote primary healthcare settings: a diagnostic test evaluation. Sex Transm Infect 2018; 94:340–5. 29748180 10.1136/sextrans-2017-053443

[24] Thammajaruk N, Ramautarsing RA, Hiransuthikul A, et al Pooled pharyngeal, rectal, and urine specimens for the point-of-care detection of *Chlamydia trachomatis* and *Neisseria gonorrhoeae* by lay providers in key population-led health services in Thailand. Pathogens 2023; 12:1268. 37887784 10.3390/pathogens12101268 PMC10609829

[25] Garrett N, Mitchev N, Osman F, et al Diagnostic accuracy of the Xpert CT/NG and OSOM trichomonas rapid assays for point-of-care STI testing among young women in South Africa: a cross-sectional study. BMJ Open 2019; 9:e026888. 10.1136/bmjopen-2018-026888 PMC6367982 30782948

[26] Speers DJ, Chua IJ, Manuel J, Marshall L. Detection of *Neisseria gonorrhoeae* and *Chlamydia trachomatis* from pooled rectal, pharyngeal and urine specimens in men who have sex with men. Sex Transm Infect 2018; 94:293–7. 29066627 10.1136/sextrans-2017-053303

[27] Badman SG, Willie B, Narokobi R, et al A diagnostic evaluation of a molecular assay used for testing and treating anorectal chlamydia and gonorrhoea infections at the point-of-care in Papua New Guinea. Clin Microbiol Infect 2019; 25:623–7. 30107282 10.1016/j.cmi.2018.08.001 PMC11005091

[28] Wilson SP, Vohra T, Goldberg J, et al Reliable rapid assay for gonorrhea and chlamydia in the emergency department. J Emerg Med 2017; 53:890–5. 29074030 10.1016/j.jemermed.2017.08.094

[29] Han Y, Shi MQ, Jiang QP, et al Clinical performance of the Xpert(®) CT/NG test for detection of *Chlamydia trachomatis* and *Neisseria gonorrhoeae*: a multicenter evaluation in Chinese urban hospitals. Front Cell Infect Microbiol 2021; 11:784610. 35047416 10.3389/fcimb.2021.784610 PMC8762110

[30] Morris SR, Bristow CC, Wierzbicki MR, et al Performance of a single-use, rapid, point-of-care PCR device for the detection of *Neisseria gonorrhoeae, Chlamydia trachomatis,* and *Trichomonas vaginalis*: a cross-sectional study. Lancet Infect Dis 2021; 21:668–76. 33242473 10.1016/S1473-3099(20)30734-9 PMC9884536

[31] Van Der Pol B, Taylor SN, Mena L, et al Evaluation of the performance of a point-of-care test for chlamydia and gonorrhea. JAMA Netw Open 2020; 3:e204819. 32407506 10.1001/jamanetworkopen.2020.4819 PMC7225902

[32] Clebak KT, Bensur J, Sell JK. Binx IO point-of-care test for *Chlamydia trachomatis* and *Neisseria gonorrhoeae* infections. Am Fam Physician 2022; 106:575–7. 36379508

[33] Harding-Esch E, Nori A, Hegazi A, et al Impact of deploying multiple point-of-care tests with a ‘sample first’ approach on a sexual health clinical care pathway: a service evaluation. Sex Transm Infect 2017; 93:sextrans-2016. 10.1136/sextrans-2016-052988 PMC5574381 28159916

[34] Van Der Pol B, Arcenas R, Boraas C, et al Sensitivity and specificity of the cobas liat CT/NG/MG nucleic acid test in a clinical laboratory setting and point-of-care location. J Clin Microbiol 2025; 63:e0070625. 41159798 10.1128/jcm.00706-25 PMC12710341

[35] Fisk KM, Derouin A, Holm G, Hicks L. Getting it right: the impact of point-of-care testing for gonorrhea and chlamydia in the urgent care setting. J Nurse Pract 2020; 16:388–93.

[36] Dawkins M, Bishop L, Walker P, et al Clinical integration of a highly accurate polymerase chain reaction point-of-care test can inform immediate treatment decisions for chlamydia, gonorrhea, and trichomonas. Sex Transm Dis 2022; 49:262–7. 34813579 10.1097/OLQ.0000000000001586

[37] May L, Ware CE, Jordan JA, et al A randomized controlled trial comparing the treatment of patients tested for chlamydia and gonorrhea after a rapid polymerase chain reaction test versus standard of care testing. Sex Transm Dis 2016; 43:290–5. 27100764 10.1097/OLQ.0000000000000438

[38] Dashler G, Maliszewski K, Saheed M, et al Real-world use of molecular point-of-care testing for Sexually Transmitted Infections (STIs) in the emergency department: why it matters for acute care management. Open Forum Infect Dis 2025; 13:ofaf749. 41488701 10.1093/ofid/ofaf749 PMC12757586

[39] Widdice LE, Hsieh YH, Silver B, Barnes M, Barnes P, Gaydos CA. Performance of the atlas genetics rapid test for *Chlamydia trachomatis* and women's attitudes toward point-of-care testing. Sex Transm Dis 2018; 45:723–7. 29771869 10.1097/OLQ.0000000000000865 PMC6179923

[40] Gettinger J, Van Wagoner N, Daniels B, Boutwell A, Van Der Pol B. Patients are willing to wait for rapid sexually transmitted infection results in a university student health clinic. Sex Transm Dis 2020; 47:67–9. 31856075 10.1097/OLQ.0000000000001083

[41] Causer LM, Kaldor JM, Fairley CK, et al A laboratory-based evaluation of four rapid point-of-care tests for syphilis. PLoS One 2014; 9:e91504. 24618681 10.1371/journal.pone.0091504 PMC3950184

[42] Trick AY, Melendez JH, Chen FE, et al A portable magnetofluidic platform for detecting sexually transmitted infections and antimicrobial susceptibility. Sci Transl Med 2021; 13:eabf6356. 33980576 10.1126/scitranslmed.abf6356 PMC8363034

[43] Ayfan AKS, Macdonald J, Irwin AD, et al Proof-of-concept, rapid, instrument-free molecular detection of *Neisseria gonorrhoeae* and ciprofloxacin susceptibility. J Antimicrob Chemother 2022; 77:2933–6. 35880750 10.1093/jac/dkac242

[44] Bandea CI, Koumans EH, Sawyer MK, et al Evaluation of the rapid BioStar optical immunoassay for detection of *Chlamydia trachomatis* in adolescent women. J Clin Microbiol 2009; 47:215–6. 19005149 10.1128/JCM.01338-08 PMC2620880

[45] Bartelsman M, de Vries HJ, Schim van der Loeff MF, Sabajo LO, van der Helm JJ. Leucocyte esterase dip-stick test as a point-of-care diagnostic for urogenital chlamydia in male patients: a multi-center evaluation in two STI outpatient clinics in Paramaribo and Amsterdam. BMC Infect Dis 2016; 16:625. 27809795 10.1186/s12879-016-1946-8 PMC5093983

[46] Chen X, Yuan W, Zhou Q, Tan Y, Wang R, Dong S. Sensitive and visual identification of *Chlamydia trachomatis* using multiple cross displacement amplification integrated with a gold nanoparticle-based lateral flow biosensor for point-of-care use. Front Cell Infect Microbiol 2022; 12:949514. 35937700 10.3389/fcimb.2022.949514 PMC9355032

[47] Gleeson B, Piton J, Mazzola L, et al Development of a novel fluorescent-based lateral flow assay for the detection of *Neisseria gonorrhoeae* at the point of care. Sex Transm Dis 2024; 51:186–91. 38412465 10.1097/OLQ.0000000000001913

[48] Peters RPH, Klausner JD, Mazzola L, et al Novel lateral flow assay for point-of-care detection of *Neisseria gonorrhoeae* infection in syndromic management settings: a cross-sectional performance evaluation. Lancet 2024; 403:657–64. 38335982 10.1016/S0140-6736(23)02240-7 PMC11246789

