# Supplementary material for: Point-of-care Tests for Chlamydia trachomatis and Neisseria gonorrhoeae: Review of the Literature
Source: Clin Infect Dis. 2026 Feb 24;82(Suppl 1):S43–51. doi: 10.1093/cid/ciaf699 (PMC13084200; doi:10.1093/cid/ciaf699)
Supplement: ciaf699_Supplementary_Data [file ciaf699_supplementary_data.zip › SupplementalFile.docx]

**Search Strategy:**

| **Database** | **Strategy** | **Run Date** | **Records** |
| --- | --- | --- | --- |
| **Medline**  **(OVID)**  **1946-** | 1. chlamydia/ OR chlamydia trachomatis/ OR Chlamydia Infections/di 2. chlamydia*.ti,ab,kf. 3. Gonorrhea/di OR Neisseria gonorrhoeae/ 4. Gonorrh*.ti,ab,kf. 5. 1 OR 2 OR 3 OR 4 6. exp Nucleic Acid Amplification Techniques/ OR exp Point-of-Care Testing/ 7. ((point-of-care ADJ5 test*) OR POC test* OR POCT* OR POC STI OR (point-of-care ADJ5 diagnos*) OR (point-of-care ADJ5 screen*) OR beside test* OR nucleic acid amplification OR NAAT* OR rapid test* OR polymerase chain reaction OR PCR*).ti,ab,kf,hw. 8. 6 OR 7 9. 5 AND 8 10. Limit 9 to english language 11. Limit 10 to yr="2009-current" 12. Limit 13 to "remove preprint records" | 01/18/2024 | 2199 |
| **Embase**  **(OVID)**  **1974-** | 1. chlamydia/ OR chlamydia trachomatis/ OR Chlamydia Infections/ 2. chlamydia*.ti,ab,kf. 3. Gonorrhea/di OR Neisseria gonorrhoeae/ 4. Gonorrh*.ti,ab,kf. 5. 1 OR 2 OR 3 OR 4 6. exp Nucleic Acid Amplification Techniques/ OR exp "Point-of-Care Testing"/ 7. ((point-of-care ADJ5 test*) OR POC test* OR POCT* OR POC STI OR (point-of-care ADJ5 diagnos*) OR (point-of-care ADJ5 screen*) OR beside test* OR nucleic acid amplification OR NAAT* OR rapid test* OR polymerase chain reaction OR PCR*).ti,ab,kf,hw. 8. 6 OR 7 9. 5 AND 8 10. Limit 9 to english language 11. Limit 10 to yr="2009-current" 12. Limit 11 to "remove preprint records" 13. Limit 12 to "remove medline records" 14. limit 13 to conference abstract status 15. 13 not 14 | 01/18/2024 | 1173  -  duplicates  =1023  unique items |
| **Cochrane Library** | 1. [mh ^chlamydia] OR [mh ^"chlamydia trachomatis"] OR [mh ^"Chlamydia Infections"] 2. chlamydia*:ti,ab 3. [mh ^Gonorrhea] OR [mh ^"Neisseria gonorrhoeae"] 4. Gonorrh*:ti,ab 5. #1 OR #2 OR #3 OR #4 6. [mh "Nucleic Acid Amplification Techniques"] OR [mh "Point-of-Care Testing"] 7. ((point-of-care:ti,ab NEAR/5 test*:ti,ab) OR ("POC" NEXT test*):ti,ab OR POCT*:ti,ab OR "POC STI":ti,ab OR (point-of-care:ti,ab NEAR/5 diagnos*:ti,ab) OR (point-of-care:ti,ab NEAR/5 screen*:ti,ab) OR ("beside" NEXT test*):ti,ab OR "nucleic acid amplification":ti,ab OR NAAT*:ti,ab OR ("rapid" NEXT test*):ti,ab OR "polymerase chain reaction":ti,ab OR PCR*:ti,ab) 8. #6 OR #7 9. #5 AND #8   yr="2009-current" | 01/18/2024 | 259  -  duplicates  =166  unique items |
| **CINAHL**  **(EBSCOHost)** | 1. (MH chlamydia) OR (MH "chlamydia trachomatis") OR (MH "Chlamydia Infections") 2. (TI chlamydia* OR AB chlamydia*) 3. (MH Gonorrhea) OR (MH "Neisseria gonorrhoeae") 4. (TI Gonorrh* OR AB Gonorrh*) 5. S1 OR S2 OR S3 OR S4 6. (MH "Nucleic Acid Amplification Techniques+") OR (MH "Point-of-Care Testing+") 7. (((TI point-of-care OR AB point-of-care) N5 (TI test* OR AB test*)) OR (TI "POC test*" OR AB "POC test*") OR (TI POCT* OR AB POCT*) OR (TI "POC STI" OR AB "POC STI") OR ((TI point-of-care OR AB point-of-care) N5 (TI diagnos* OR AB diagnos*)) OR ((TI point-of-care OR AB point-of-care) N5 (TI screen* OR AB screen*)) OR (TI "beside test*" OR AB "beside test*") OR (TI "nucleic acid amplification" OR AB "nucleic acid amplification") OR (TI NAAT* OR AB NAAT*) OR (TI "rapid test*" OR AB "rapid test*") OR (TI "polymerase chain reaction" OR AB "polymerase chain reaction") OR (TI PCR* OR AB PCR*)) 8. S6 OR S7 9. S5 AND S8   **Limiters** - Publication Date: 20090101-20241231; English Language; Exclude MEDLINE records | 01/18/2024 | 199  -  duplicates  =90  unique items |
| **Scopus** | ((INDEXTERMS(chlamydia) OR INDEXTERMS("chlamydia trachomatis") OR INDEXTERMS("Chlamydia Infections")) OR (TITLE-ABS(chlamydia*)) OR (INDEXTERMS(Gonorrhea) OR INDEXTERMS("Neisseria gonorrhoeae")) OR (TITLE-ABS(Gonorrh*))) AND ((INDEXTERMS("Nucleic Acid Amplification Techniques") OR INDEXTERMS("Point-of-Care Testing")) OR (TITLE-ABS((point-of-care W/5 test*) OR "POC test*" OR POCT* OR "POC STI" OR (point-of-care W/5 diagnos*) OR (point-of-care W/5 screen*) OR "beside test*" OR "nucleic acid amplification" OR NAAT* OR "rapid test*" OR "polymerase chain reaction" OR PCR*))) AND NOT INDEX(medline)  Limit 2009-current ; English | 01/18/2024 | 924  -  duplicates  =259  unique items |

Notes: Duplicates were identified using the Endnote automated "find duplicates" function with preference set to match on title, author and year, and removed from your Endnote library. There will likely be additional duplicates found that Endnote was unable to detect.
